# P-625. Deaths Associated with Laboratory-confirmed COVID-19-associated Hospitalizations, Including In-hospital and Post-discharge Deaths, COVID-NET, March 2020 – September 2023

**DOI:** 10.1093/ofid/ofaf695.838

**Published:** 2026-01-11

**Authors:** Fiona P Havers, Kadam Patel, Jennifer Milucky, Jeremy L Roland, Breanna Kawasaki, Julie Plano, Lucy S Witt, Val Tellez Nunez, Erica Martin, Adrienne Domen, Fiona Keating, Katherine St. George, Melissa Sutton, Christopher A Taylor

**Affiliations:** Centers for Disease Control and Prevention, Atlanta, Georgia; Centers for Disease Control and Prevention, Atlanta, Georgia; CDC, Atlanta, Georgia; California Emerging Infections Program, Oakland, California; Colorado Department of Public Health and Enviornment, Denver, Colorado; Connecticut Emerging Infections Program, Yale School of Public Health, New Haven, Connecticut; Emory University, Atlanta, Georgia; Michigan Department of Health and Human Services, Lansing, Michigan; Minnesota Department of Health, Saint Paul, Minnesota; University of New Mexico, Emerging Infections Program, Albuquerque, New Mexico; New York State Department of Health, Albany, New York; University of Rochester Medical Center, Rochester, New York; Public Health Division, Oregon Health Authority, Portland, Oregon; Centers for Disease Control and Prevention, Atlanta, Georgia

## Abstract

**Background:**

Deaths from COVID-19 in the US have been tracked using death certificates with COVID-19 listed as a cause of death (COD). However, non-specific CODs or complications of COVID-19, such as myocardial infarction, may be listed as COD for those who died because of COVID-19. We aimed to describe trends in all-cause mortality associated with COVID-19-associated hospitalization and how those deaths are coded on death certificates. Data from the COVID-19-Associated Hospitalization Surveillance Network (COVID-NET) were used to describe in-hospital and post-discharge mortality from March 2020–September 2023.

**Table.**
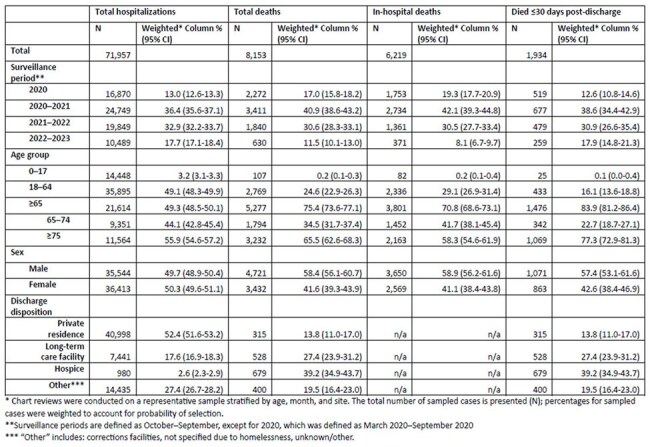
Demographic characteristics of patients with COVID-19-associated hospitalizations, March 2020-September 2023

**Figure 1.**
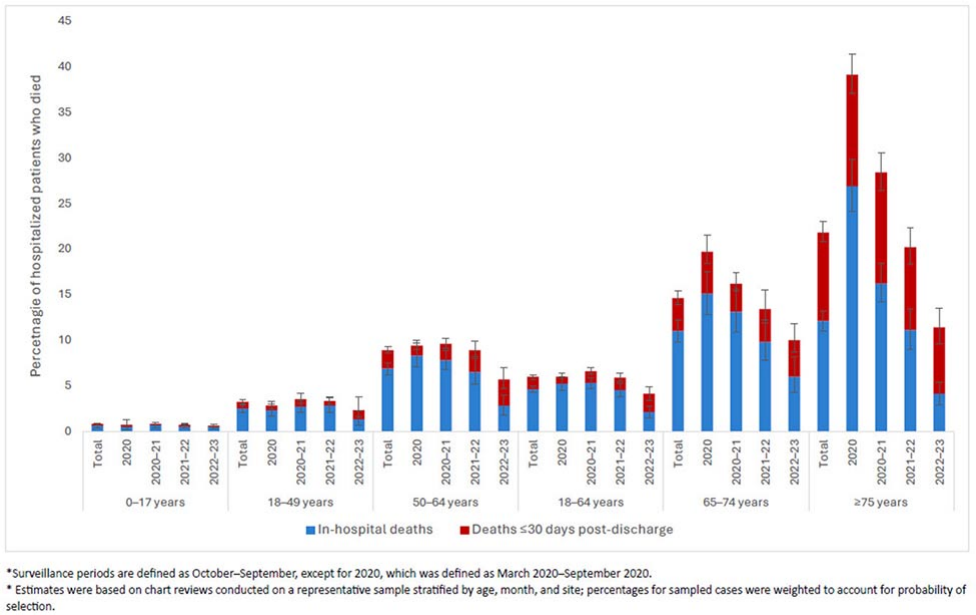
Percentage of patients with COVID-19-associated hospitalizations who died in-hospital or ≤30 days of discharge, by age group and surveillance period,* COVID-NET, March 2020-September 2023.

**Methods:**

COVID-NET is a population-based surveillance system in 12 states that captures laboratory-confirmed COVID-19-associated hospitalizations of any patient residing in the catchment areas with a positive SARS-CoV-2 test during hospitalization or ≤ 14 days prior to admission. Chart reviews were conducted on a random sample stratified by age, site and month; percentages were weighted to account for the probability of selection. Hospitalizations were linked to death certificates to identify those who died ≤ 30 days of hospital discharge; the proportion with COVID-19 as a COD was described. Logistic regression was used to assess trends.

**Figure 2.**
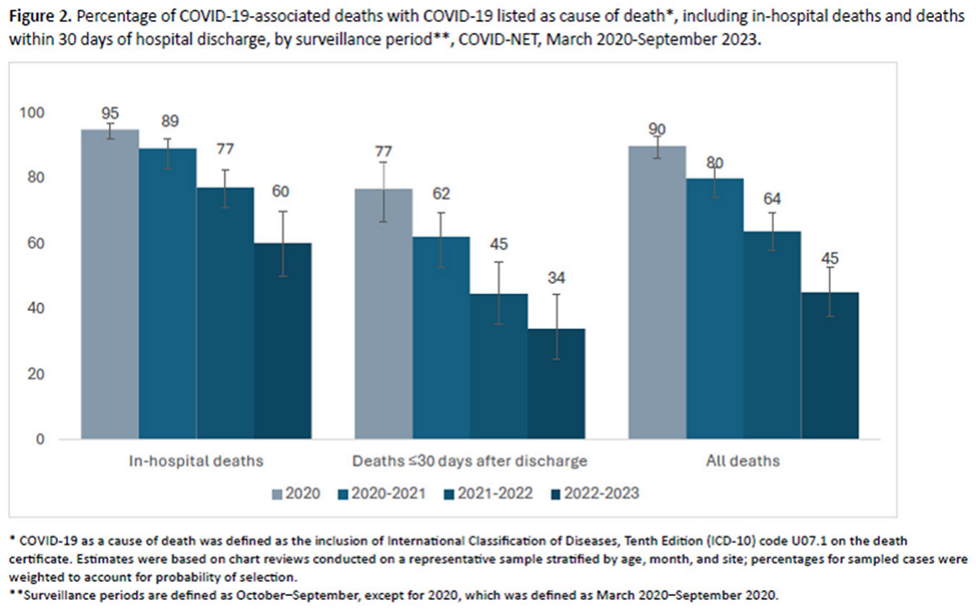
Percentage of COVID-19-associated deaths with COVID-19 listed as cause of death*, including in-hospital deaths and deaths within 30 days of hospital discharge, by surveillance period**, COVID-NET, March 2020-September 2023.

**Results:**

482,734 COVID-NET hospitalizations were identified during the study period; 71,729 (14.9%) had chart review. Among these, 12% were in patients who died; 40.9% (95% confidence interval (CI): 38.6%–43.2%) of deaths occurred in October 2020-September 2021 (Table). The proportion of hospitalized patients who died decreased over time in all age groups (p< 0.001) (Figure 1). 65.5% (95% CI: 62.6%–68.3%) of all deaths occurred among those ≥75 years, 44.6% (95% CI: 40.9%–48.4%) of which occurred post-discharge (Figure 1). This group also comprised 77.3% (95% CI: 72.9%–81.3%) of post-discharge deaths. Among in-hospital deaths, the proportion with a COVID-19 COD decreased from 95% (95% CI: 92%–97%) in 2019–2020 to 60% (95% CI: 50%–70%) in 2022–2023 (Figure 2).

**Conclusion:**

A substantial proportion of COVID-19-associated deaths occurred after discharge, especially in older adults, who accounted for most deaths. Ascertainment based on COVID-19 as a COD may underestimate COVID-19-associated deaths.

**Disclosures:**

Lucy S. Witt, MD, MPH, Merck & Co: Grant/Research Support Val Tellez Nunez, MPH, Michigan Department of Health and Human Services, CSTE Federal Grant: Grant/Research Support Melissa Sutton, MD, MPH, Centers for Disease Control and Prevention Emerging Infections Program: Grant/Research Support

